# Radiomic hemorrhage roundness predicts outcome beyond the ICH score in deep intracerebral hemorrhage with IVH

**DOI:** 10.1007/s00415-026-13875-1

**Published:** 2026-05-22

**Authors:** Alexandru Guranda, Johannes Wach, Sebastian Lehmann, Felix Arlt, Alim Emre Basaran, Tim Wende, Erdem Güresir, Martin Vychopen

**Affiliations:** Department of Neurosurgery, University Hospital Leipzig, Liebigstrasse 20, 04103 Leipzig, Germany

**Keywords:** Intracerebral hemorrhage, Basal ganglia hemorrhage, Intraventricular hemorrhage, Hematoma morphology, ICH score

## Abstract

**Background:**

Accurate prognostication in deep supratentorial intracerebral hemorrhage (ICH) remains challenging. Hematoma volume is a key determinant of outcome and a component of the ICH Score. Whether radiomics-derived shape features provide prognostic information beyond volumetric burden and clinical risk scores remains unclear. We investigated the association between radiomic shape characteristics of basal ganglia hemorrhage (BGH) and intraventricular hemorrhage (IVH) with in-hospital mortality and unfavorable functional outcome.

**Methods:**

We retrospectively analyzed 50 patients with deep supratentorial ICH and IVH. Semi-automated segmentations were used to extract radiomic features (roundness, elongation, flatness, Feret diameter, surface area) and volumetric parameters. Logistic regression models were constructed for in-hospital mortality and unfavorable functional outcome (mRS 5–6 at discharge; secondary definition mRS 4–6), with collinearity assessment guiding feature selection. Model discrimination was evaluated using receiver operating characteristic curves with DeLong comparisons. Secondary models incorporated the ICH Score. Calibration and internal validation were assessed using the Hosmer–Lemeshow test and bootstrap resampling (1000 iterations).

**Results:**

For in-hospital mortality, BGH roundness showed discrimination comparable to BGH volume (AUC 0.742 vs 0.738). In multivariable analysis, lower roundness remained independently associated with mortality after adjustment for the ICH Score (OR 0.20 per 0.1 increase; 95% CI 0.04–0.66; *p* = 0.021), whereas volume was no longer significant. The combined model achieved an AUC of 0.866 versus 0.813 for the ICH Score (DeLong *p* = 0.28). IVH-derived shape parameters were not associated. For unfavorable outcome, BGH volume remained strongest predictor.

**Conclusions:**

Radiomic hemorrhage roundness may provide complementary prognostic information beyond hematoma volume and the ICH Score in deep ICH with IVH.

**Supplementary Information:**

The online version contains supplementary material available at 10.1007/s00415-026-13875-1.

## Introduction

Spontaneous intracerebral hemorrhage (ICH) is associated with high in-hospital mortality and substantial long-term disability [16]. Accurate early prognostication remains a central challenge in clinical practice, as it directly influences treatment decisions. Established predictors of outcome include age, admission neurological status, hematoma volume, and the presence of intraventricular hemorrhage [19]. These variables are incorporated into widely used clinical scores such as the ICH Score [7].

Hematoma volume has consistently emerged as one of the strongest predictors of mortality and functional outcome [12]. Volume reflects the overall hemorrhagic burden and correlates with mass effect and tissue destruction. However, radiologically, hemorrhages of similar size often differ substantially in their geometric configuration. Such differences may reflect variations in bleeding dynamics, spatial expansion patterns, or secondary disruption of surrounding tissue [10, 11]. These structural aspects are not fully captured by volume-based metrics alone, as incorporated in the ICH score, where volume is dichotomized at a threshold of 30 cm^3^ based on the ABC/2 formula, without any further refinement or adjustment [7].

Quantitative imaging approaches now allow objective characterization of lesion morphology beyond simple volumetric assessment [8]. Radiomic shape descriptors, including roundness, elongation, and surface characteristics, provide reproducible measures of hemorrhage geometry [3]. While radiomic methods have been increasingly investigated in oncologic imaging and ischemic stroke, data on their prognostic relevance in spontaneous ICH remain limited [8, 20]. In particular, it remains unclear whether morphologic shape characteristics provide prognostic value beyond established clinical predictors such as hematoma volume and the ICH Score.

Deep supratentorial ICH, especially involving the basal ganglia, represents a relatively homogeneous anatomical subgroup in which mass effect and intraventricular extension frequently coexist [4]. This anatomical subgroup allows a focused evaluation of whether geometric features of hemorrhage are independently associated with outcome. By restricting the cohort to patients with concomitant intraventricular hemorrhage, we aimed to further reduce clinical heterogeneity and focus on a high-risk subgroup in which both parenchymal and ventricular components contribute to outcome.

In this study, we evaluated the association between radiomic shape characteristics of BGH and clinical outcome in patients with deep supratentorial ICH. We hypothesized that hemorrhage roundness, as a quantitative measure of geometric regularity, would be independently associated with in-hospital mortality and provide prognostic information beyond hematoma volume as represented in the dichotomized ICH Score.

## Methods

### Study design and patient population

This retrospective single-center cohort study included adult patients admitted between 2020 and January 2026 with spontaneous deep supratentorial ICH. Inclusion required a primary BGH with concomitant IVH on initial non-contrast computed tomography (CT). Patients with secondary hemorrhage due to vascular malformations, intracranial tumors, trauma, or hemorrhagic transformation of ischemic stroke were excluded. The study was approved by the Ethics Committee of Leipzig University (reference number: 073/26-ek) in accordance with institutional and national guidelines and regulations. The requirement for informed consent was waived due to the retrospective study design and anonymized data analysis.

### Clinical data collection and outcome definition

Baseline demographic and clinical variables were extracted from electronic medical records, including age, sex, admission Glasgow Coma Scale (GCS), and variables required to calculate the ICH Score according to established criteria [7]. The ICH Score was derived from admission clinical and radiologic parameters. The primary endpoint was in-hospital mortality. The secondary endpoint was unfavorable functional outcome at discharge, defined as modified Rankin Scale (mRS) score 5–6. Functional status was determined based on documented neurological assessment at discharge. An additional exploratory analysis was performed using an alternative definition of unfavorable outcome (mRS 4–6).

### Imaging acquisition and hematoma segmentation

**Fig. 1:**
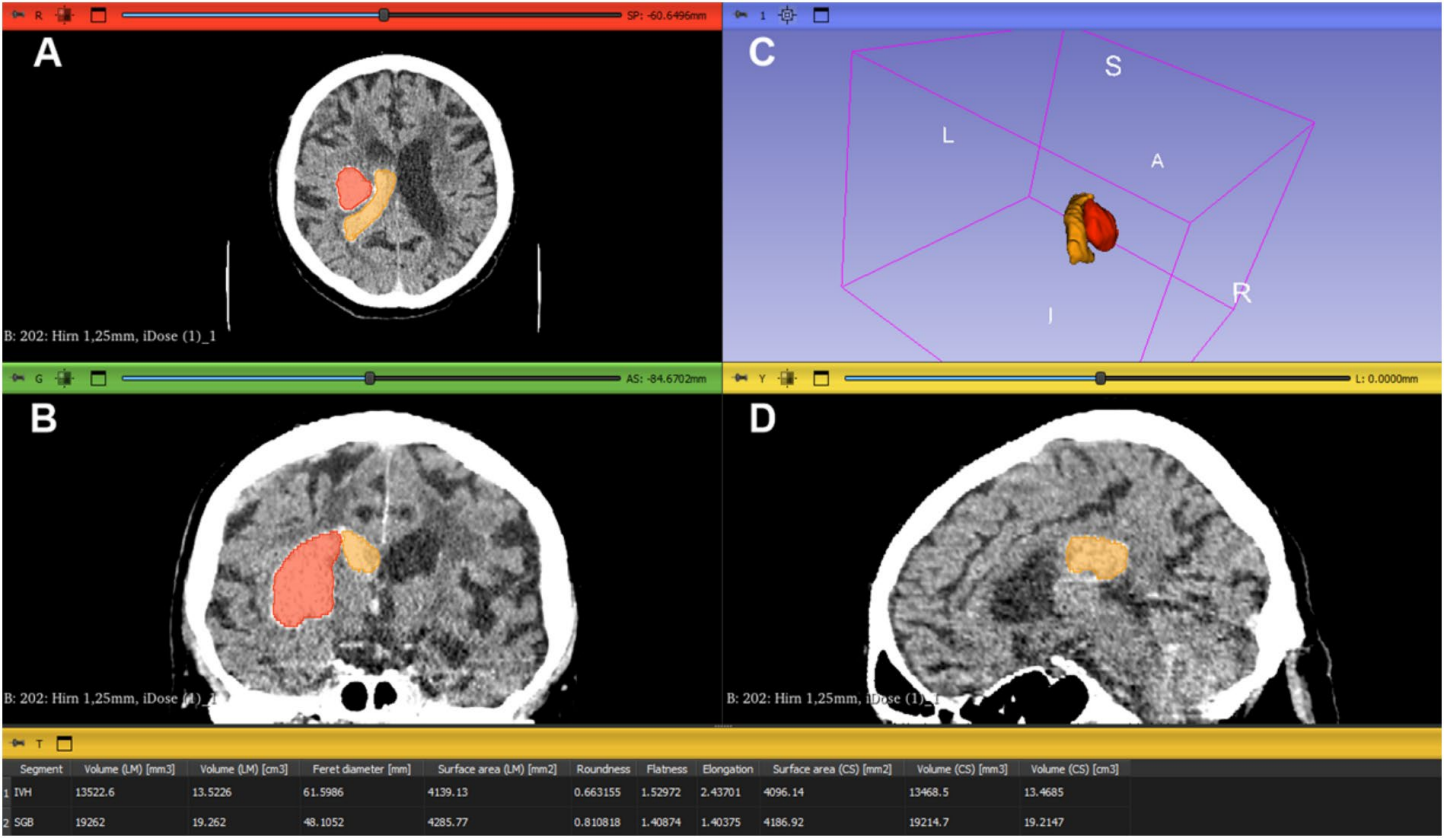
Representative segmentation of deep supratentorial intracerebral hemorrhage with intraventricular extension. **A** Axial and **B** coronal non-contrast CT images demonstrating basal ganglia hemorrhage (BGH, red) and intraventricular hemorrhage (IVH, orange). **C** 3D reconstruction of the segmented hemorrhage compartments. **D** Sagittal CT slice illustrating the spatial configuration of the hemorrhage

All patients underwent non-contrast CT imaging at admission according to institutional stroke protocols. Hematoma segmentation was performed using 3D Slicer (version 5.6.2; www.slicer.org), as illustrated in Fig. 1. A semi-automated threshold-based approach was applied, followed by manual refinement where necessary to ensure anatomical accuracy. Separate segmentations were generated for BGH and IVH, enabling independent volumetric and morphologic analyses of both compartments. Volumes were calculated directly from the segmented regions and reported in cubic millimeters and cubic centimeters.

### Radiomic shape feature extraction

Shape-based radiomic features were extracted from segmented hemorrhage volumes using geometric descriptors implemented in 3D Slicer. Roundness was defined as a measure of similarity to a sphere, calculated as the ratio between the surface area of a sphere derived from the Feret diameter and the actual surface area of the segmented object, with a value of 1 indicating a perfect sphere. Representative examples of hematomas with high and low roundness are provided in Supplementary Figure S1. Elongation and flatness were calculated from the principal moments of inertia of the segmented volume, reflecting the relative proportions of the principal axes and deviations from isotropic geometry. The Feret diameter was defined as the diameter of the smallest sphere enclosing the entire segmented volume. Surface area corresponded to the total area of the three-dimensional hemorrhage boundary. To limit dimensionality and reduce the risk of overfitting, only shape features were analyzed. These included roundness, elongation, flatness, Feret diameter, and surface area. No intensity- or texture-based features were considered. For regression analyses, roundness values were scaled by a factor of 10 to facilitate interpretation of regression coefficients, corresponding to changes per 0.1 unit on the original scale.

### Statistical analysis

Continuous variables are reported as mean ± standard deviation or median (IQR), as appropriate; categorical variables as counts and percentages. Associations with in-hospital mortality were evaluated using logistic regression, reporting odds ratios (ORs) with 95% confidence intervals (CIs). Correlations between volumetric and morphologic parameters were assessed using Pearson coefficients. Multicollinearity was examined using variance inflation factors, and collinear variables were not included simultaneously in multivariable models. Two prespecified multivariable models were constructed. The first included BGH roundness and BGH volume to assess whether geometric characteristics were independently associated with mortality beyond volumetric burden. The second model additionally incorporated the ICH Score to evaluate incremental prognostic value. Although the ICH Score includes dichotomized hematoma volume (> 30 cm^3^), continuous BGH volume was modeled to retain granularity [7]. Given the limited number of outcome events (*n* = 17), the number of predictors was restricted. Additional sensitivity analyses were performed including IVH volume and sex as covariates. For exploratory analyses, patients were stratified into high and low BGH roundness groups based on the median value. Model discrimination was assessed using receiver operating characteristic (ROC) analysis with comparison of AUCs by DeLong’s test. Calibration was evaluated using the Hosmer–Lemeshow test. Internal validation was performed by bootstrap resampling (1000 iterations). Analogous analyses were conducted for unfavorable functional outcome (mRS 5–6; mRS 4–6). All analyses were performed using R (version 4.4.2). A two-sided *p* < 0.05 was considered statistically significant.

## Results

### Cohort characteristics

A total of 50 patients with BGH and concomitant IVH were included. In-hospital mortality occurred in 17 patients (34%), and unfavorable functional outcome at discharge (mRS 5–6) was observed in 28 patients (56%). Baseline demographic, clinical, and imaging characteristics are summarized in Table 1. The median age was 71.0 years (IQR 62.0–78.0), and 29 patients (58%) were male. The median admission Glasgow Coma Scale score was 6.0 (IQR 3.0–11.0). The median ICH Score was 3 (IQR 2–4). Median length of stay was 13 days (IQR 7–23), ranging from 1 to 61 days. Selected comorbidities were present in the cohort, including diabetes (22%), heart failure (26%), chronic obstructive pulmonary disease (10%), and malignancy (12%), while dementia and peripheral arterial disease were less frequent (each 6%).

**Table 1:** Baseline demographic, clinical, and imaging characteristics of patients with BGH and concomitant IVH. Continuous variables are presented as median (IQR), and categorical variables as number (percentage)

Characteristic	*N* = 50
Age	71 (62–78)
Sex – Female	21 (42%)
Sex – Male	29 (58%)
Admission GCS	6 (3–11)
ICH Score (median, IQR)	3 (2–4)
ICH Score—1	4 (8%)
ICH Score – 2	16 (32%)
ICH Score – 3	17 (34%)
ICH Score – 4	12 (24%)
ICH Score – 5	1 (2%)
BGH volume (cm^3^)	16.4 (8.5–40.7)
IVH volume (cm^3^)	8.7 (4–22.8)
In-hospital mortality	17 (34%)
Unfavorable outcome (mRS 5–6)	28 (56%)
Unfavorable outcome (mRS 4–6)	41 (82%)

*GCS* Glasgow Coma Scale, *ICH* intracerebral hemorrhage, *IVH* intraventricular hemorrhage; *mRS* modified Rankin scale, *BGH* basal ganglia hemorrhage

### Univariable screening of morphologic parameters

**Fig. 2:**
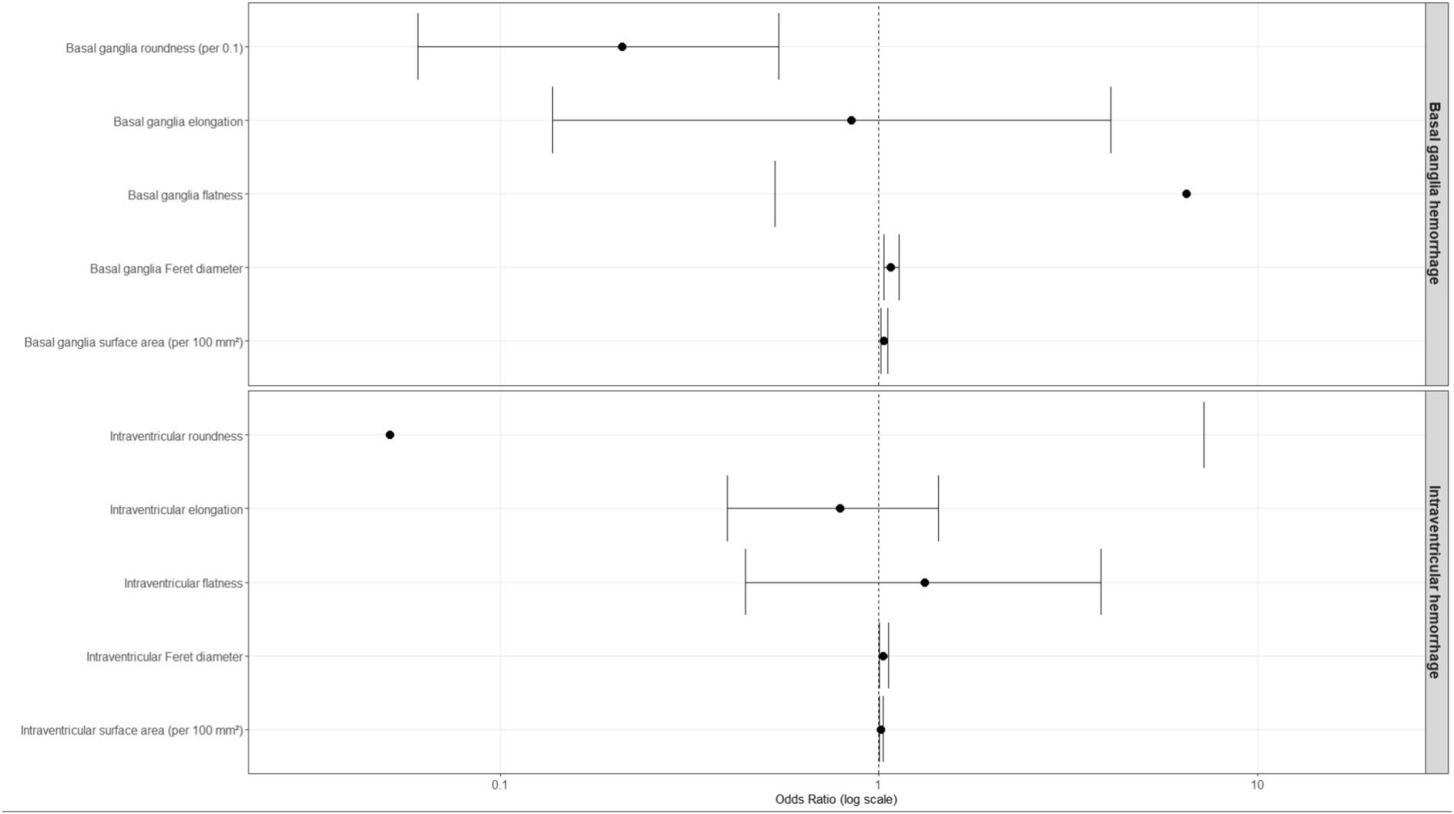
Univariable associations of morphologic radiomic parameters with in-hospital mortality. Forest plot displaying odds ratios and 95% confidence intervals from univariable logistic regression analyses of shape-derived radiomic features of basal ganglia hemorrhage and intraventricular hemorrhage. The vertical dashed line indicates an odds ratio of 1. Surface area variables are scaled per 100 mm^2^

All extracted shape parameters of the BGH and IVH were evaluated in univariable logistic regression models for in-hospital mortality. The full set of univariable associations is displayed in Fig. 2.

**Fig. 3:**
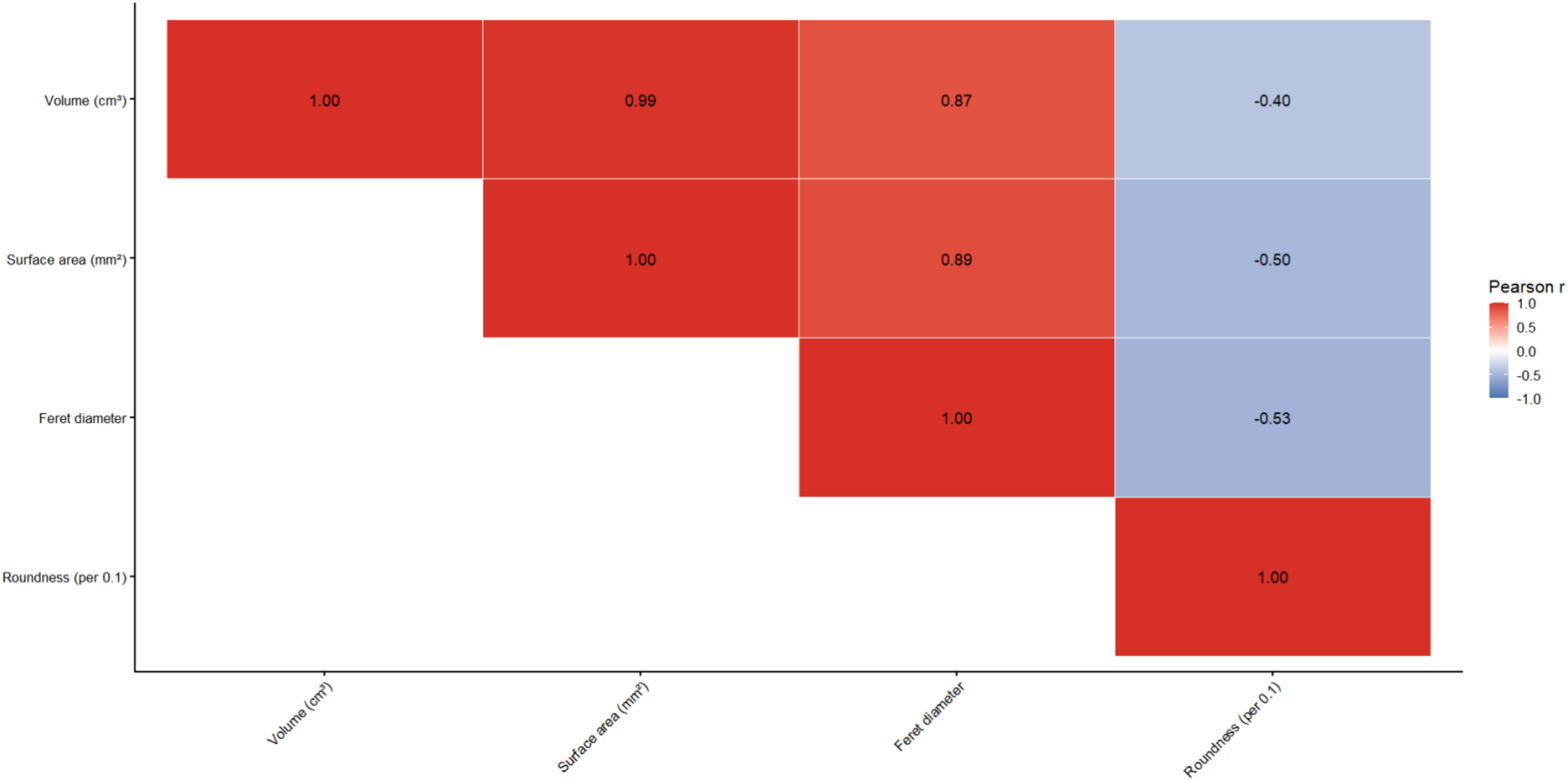
Correlation matrix of basal ganglia morphologic parameters. Pearson correlation matrix of volumetric and geometric descriptors of BGH. Strong correlations were observed between volume, surface area, and Feret diameter, whereas roundness showed only moderate correlation with size-related parameters

Among BGH-derived features, roundness (OR 0.21 per 0.1 increase, 95% CI 0.06–0.54; *p* = 0.004), Feret diameter (OR 1.07, 95% CI 1.03–1.13; *p* = 0.002), and surface area (OR 1.03 per 100 mm^2^, 95% CI 1.01–1.06; *p* = 0.005) were significantly associated with mortality, whereas elongation and flatness were not. IVH Feret diameter (OR 1.03, 95% CI 1.00–1.06; *p* = 0.044) and IVH surface area (OR 1.01 per 100 mm^2^, 95% CI 1.00–1.03; *p* = 0.032) also demonstrated significant associations. Given the conceptual overlap between volumetric and geometric size descriptors, correlation analysis was performed. As shown in Fig. 3, BGH surface area (*r* = 0.99) and BGH Feret diameter (*r* = 0.87) demonstrated very strong correlation with BGH volume, whereas roundness showed only moderate correlation (*r* = − 0.40). Variance inflation factor analysis confirmed relevant multicollinearity for surface area (VIF 4.92) and Feret diameter (VIF 5.13), while roundness exhibited low collinearity (VIF 1.39). Based on these findings, roundness was selected as the primary morphologic parameter for multivariable modeling. Baseline characteristics stratified by high versus low BGH roundness are provided in Supplementary Table S1. No significant differences in key clinical or radiological variables were observed between groups.

### Morphologic model and in-hospital mortality

In univariable analysis, lower BGH roundness (OR 0.21 per 0.1 increase; 95% CI 0.06–0.54; *p* = 0.004) and larger BGH volume (OR 1.04 per cm^3^ increase; 95% CI 1.01–1.08; *p* = 0.010) were significantly associated with mortality. In a multivariable model including BGH roundness and BGH volume, both parameters remained independently associated with mortality (Table 2). Lower roundness was associated with increased mortality (OR 0.21; 95% CI 0.05–0.65; *p* = 0.016), and larger hematoma volume remained significant (OR 1.04; 95% CI 1.01–1.08; *p* = 0.028).

**Table 2:** Logistic regression models for in-hospital mortality. Univariable and multivariable analyses evaluating associations between BGH morphologic radiomic features and mortality. Odds ratios are reported with 95% confidence intervals

Variable	OR	95% CI	p—Value
Morphologic model			
BGH roundness (per 0.1 increase)	0.21	0.05–0.65	0.016
BGH volume (per cm^3^)	1.04	1.01–1.08	0.028
Full model (incl. ICH Score)			
ICH Score (per point)	3.42	1.29–11.73	0.025
BGH volume (per cm^3^)	1.02	0.98–1.06	0.308
BGH roundness (per 0.1 increase)	0.20	0.04–0.66	0.021

*CI* confidence interval, *ICH* intracerebral hemorrhage, *OR* odds ratio, *BGH* basal ganglia hemorrhage. The morphologic model includes BGH roundness and BGH volume. The fully adjusted model additionally includes the ICH Score

**Fig. 4:**
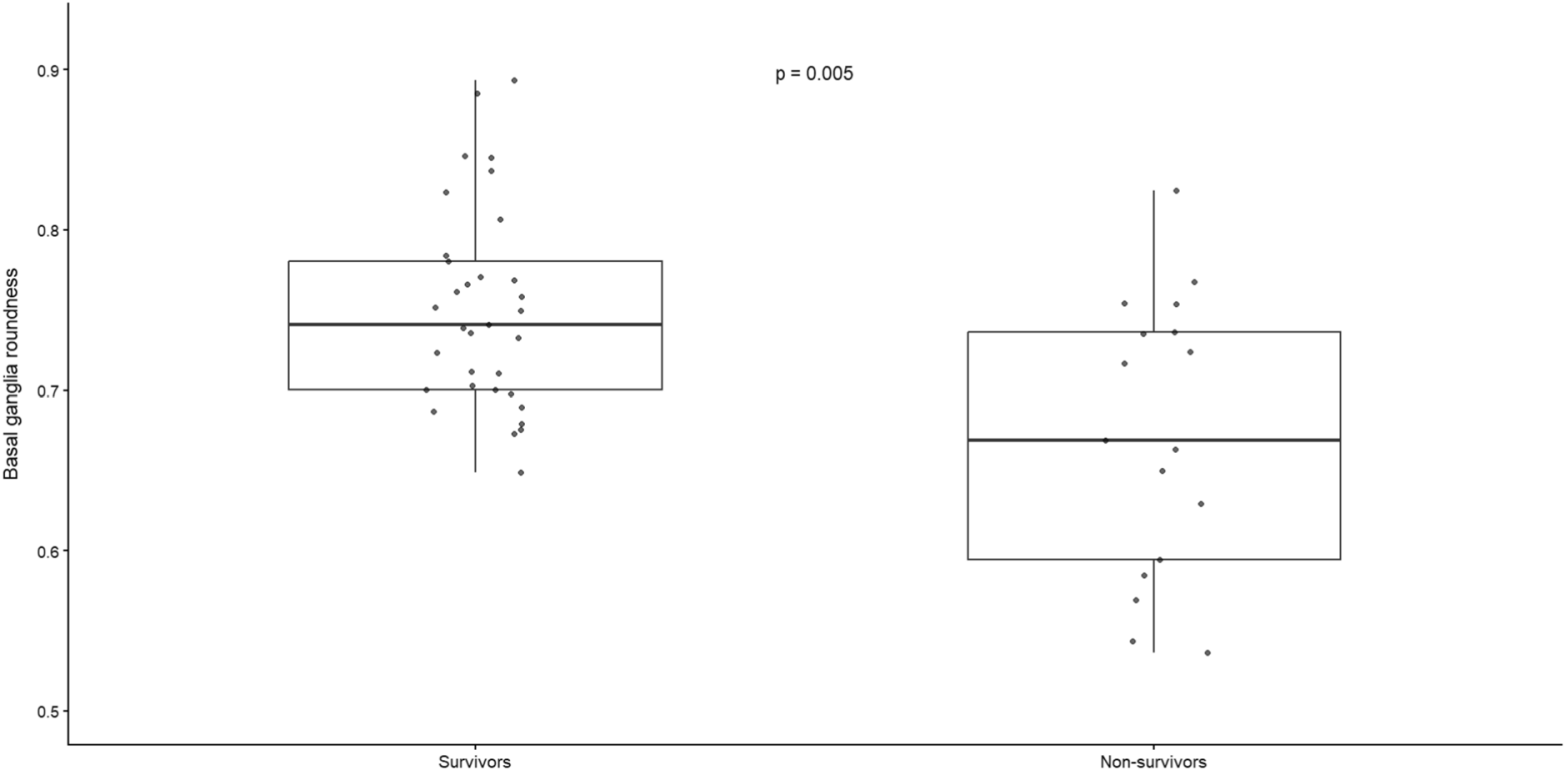
BGH roundness stratified by in-hospital mortality. Distribution of BGH in survivors and non-survivors. Boxes represent the interquartile range with the median; dots represent individual observations

The distribution of BGH roundness stratified by survival status is illustrated in Fig. 4, demonstrating significantly lower roundness values among non-survivors (Wilcoxon *p* = 0.005).

### Prognostic contribution beyond the ICH score

**Fig. 5:**
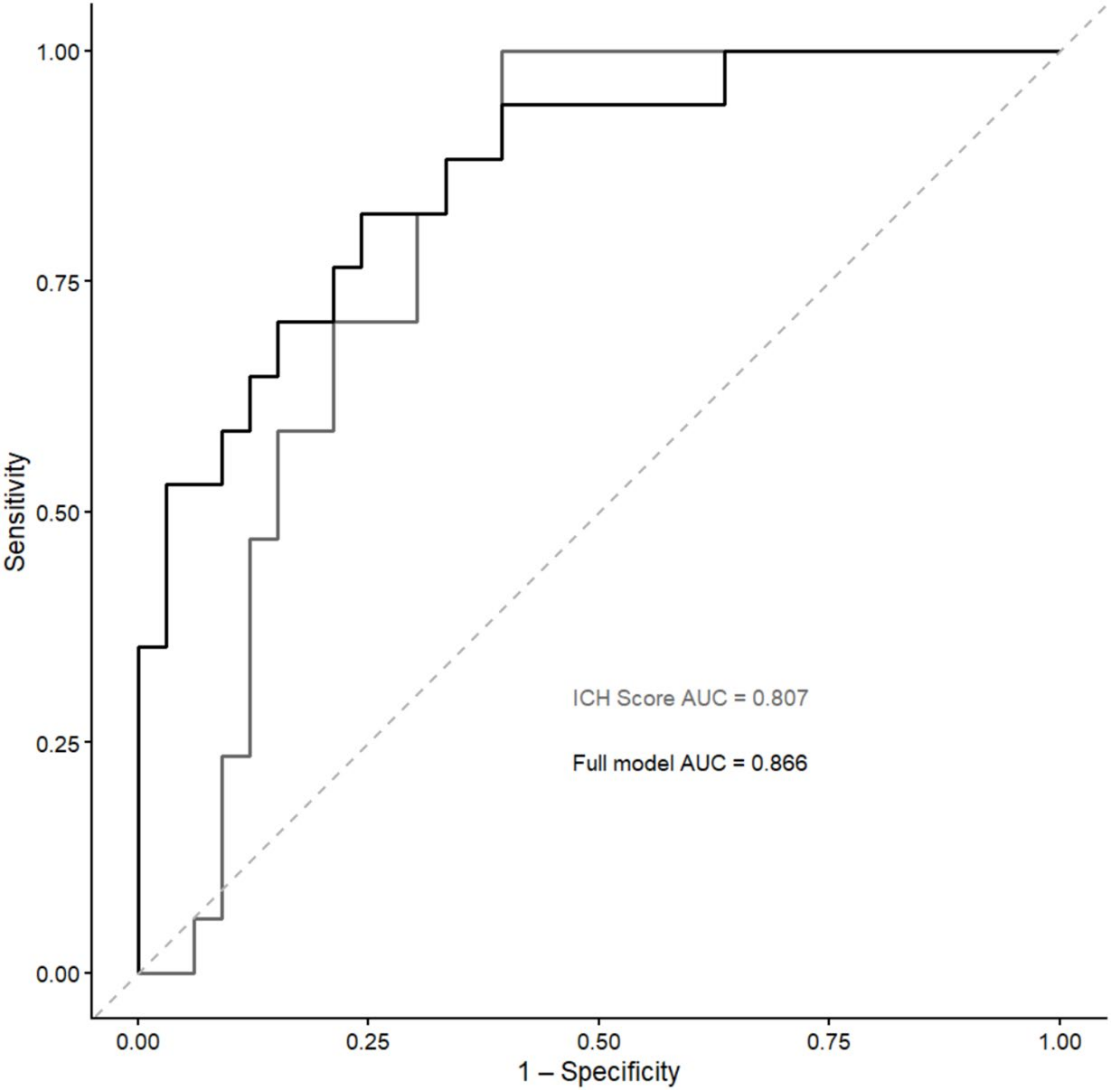
Receiver operating characteristic (ROC) curves comparing discrimination for in-hospital mortality between the ICH Score alone and the combined model including the ICH Score, BGH volume, and BGH roundness. Area under the curve (AUC) values are indicated

The ICH Score was strongly associated with mortality in univariable analysis (OR 4.15 per point; 95% CI 1.87–11.32; *p* = 0.002). In the fully adjusted model including ICH Score, BGH volume, and BGH roundness, both the ICH Score and roundness remained independently associated with mortality, whereas BGH volume lost statistical significance (Table 2). The ICH Score alone demonstrated good discrimination for mortality (AUC 0.813). The combined model including ICH Score, BGH volume, and BGH roundness yielded an AUC of 0.866 (Fig. 5). Although this represented a numerical increase, the difference did not reach statistical significance (DeLong *p* = 0.28).

**Fig. 6:**
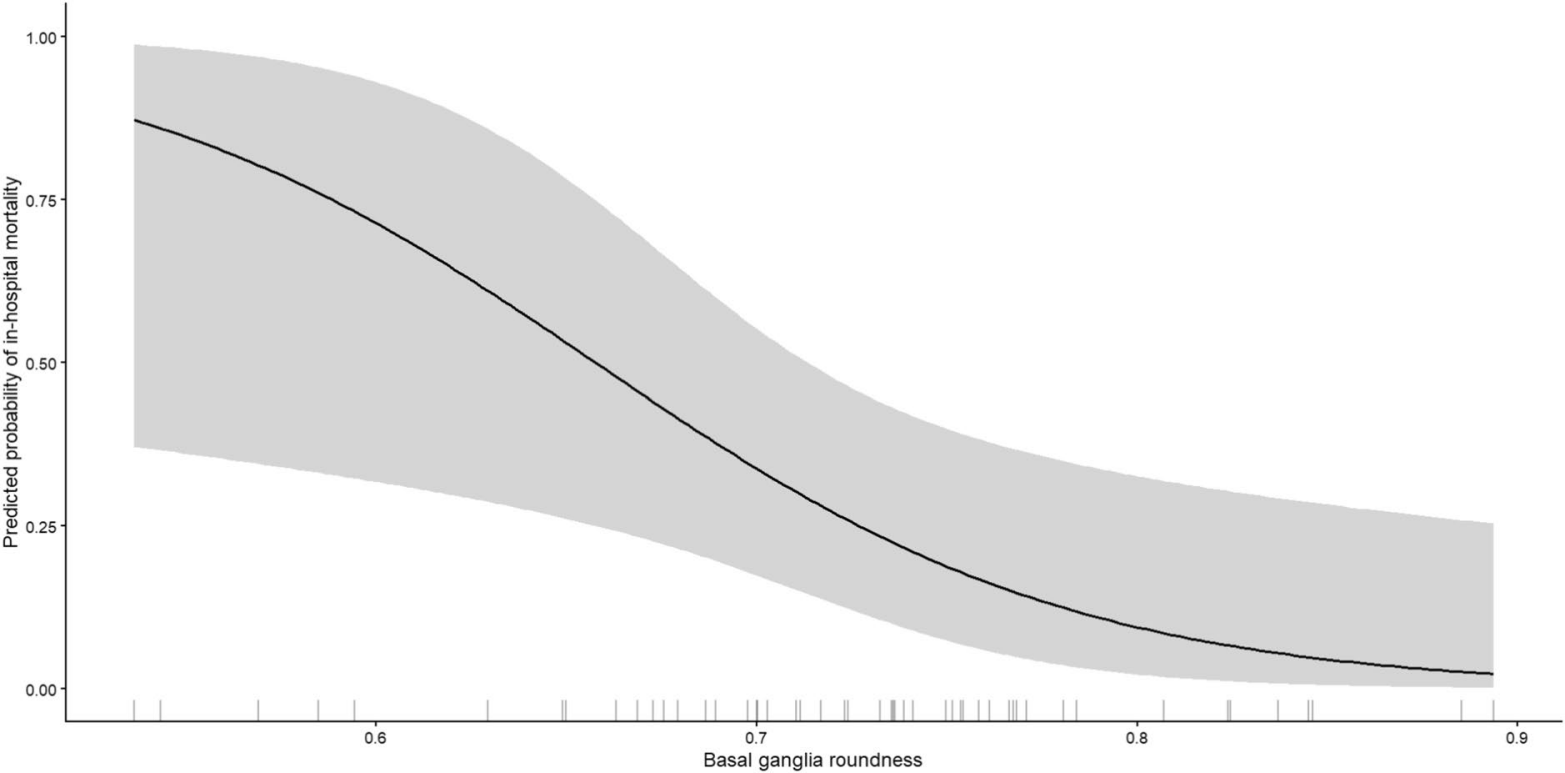
Adjusted predicted probability of in-hospital mortality according to BGH roundness. Predicted probability curve derived from the multivariable logistic regression model including the ICH Score and BGH volume. The shaded area represents the 95% confidence interval. Tick marks along the x-axis indicate observed patient values

The adjusted predicted probability of mortality across the range of basal ganglia roundness is shown in Fig. 6, demonstrating a steep increase in mortality risk with decreasing roundness, independent of ICH Score and hematoma volume. Model performance was supported by a Nagelkerke R^2^ of 0.52. Hosmer–Lemeshow testing indicated good calibration (*χ*^2^ = 4.14, *p* = 0.84). Internal validation using bootstrap resampling (1000 iterations) confirmed coefficient stability, with the percentile-based 95% confidence interval for the roundness coefficient not crossing zero (− 4.45 to − 0.39). In an additional sensitivity analysis including IVH volume and sex as covariates, the association between BGH roundness and mortality remained robust (OR 0.21 per 0.1 increase, 95% CI 0.03–0.74). IVH volume was also independently associated with mortality, whereas BGH volume remained non-significant. Sex was not independently associated with mortality and did not materially alter the effect estimates.

### Functional outcome

**Fig. 7:**
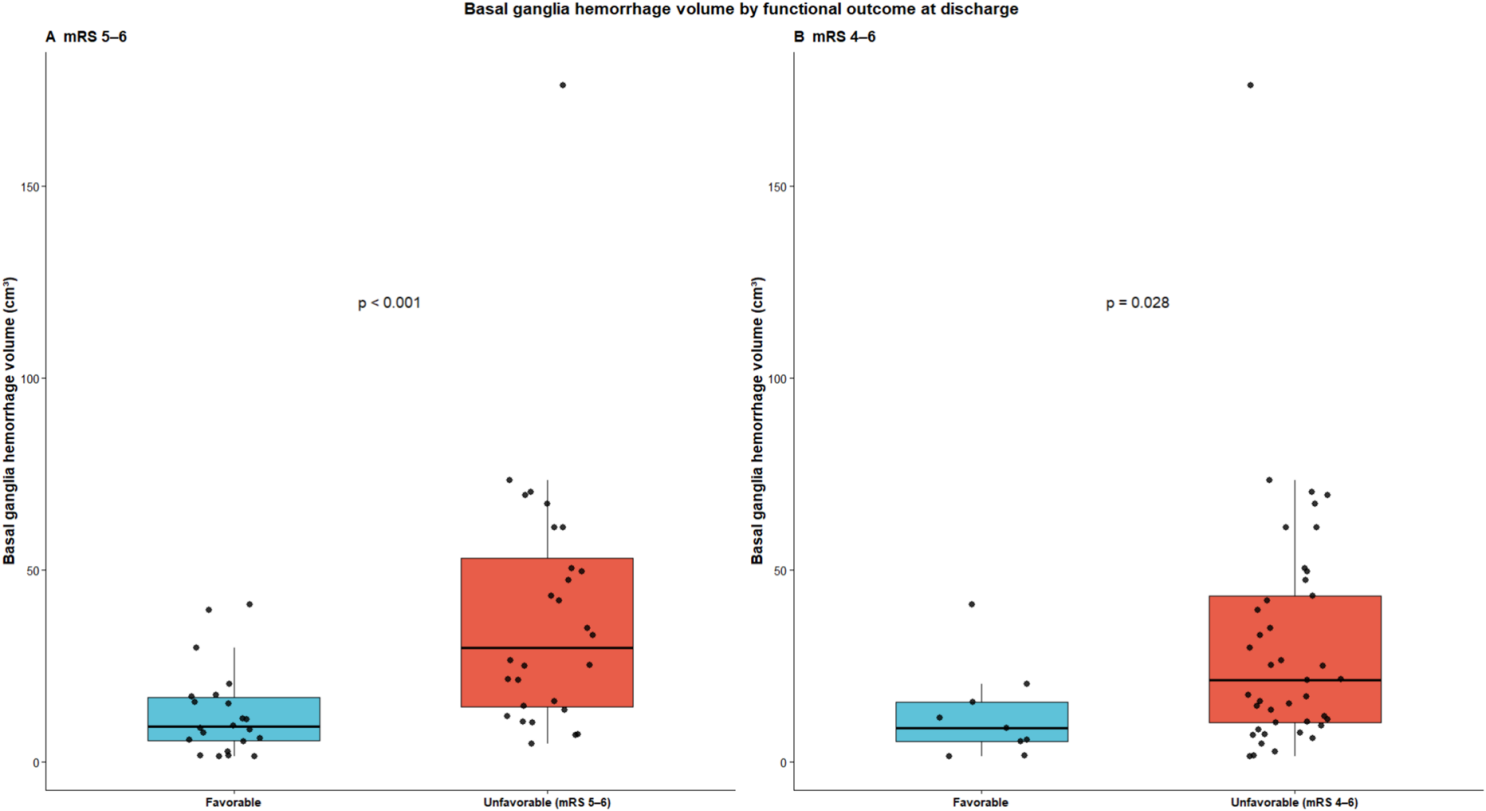
Basal ganglia hemorrhage volume by functional outcome at discharge using two outcome definitions. **A** Unfavorable outcome defined as mRS 5–6. **B** Unfavorable outcome defined as mRS 4–6. Patients with unfavorable outcome exhibited larger hematoma volumes compared with those with favorable outcome. Boxes represent the interquartile range with the median; dots represent individual observations

For unfavorable functional outcome at discharge (mRS 5–6), larger BGH volume was significantly associated with poor outcome (OR 1.08 per cm^3^ increase; *p* = 0.003), whereas roundness did not reach statistical significance (OR 0.48 per 0.1 increase; 95% CI 0.20–1.02; *p* = 0.073). The distribution of hematoma volume stratified by functional outcome is illustrated in Fig. 7, demonstrating substantially larger volumes among patients with unfavorable outcome compared with those with favorable outcome at discharge. In an additional analysis using mRS 4–6 as the definition of unfavorable outcome, a similar distribution pattern was observed. However, in multivariable analysis, neither roundness nor hematoma volume was independently associated with outcome after adjustment for the ICH Score.

## Discussion

In this retrospective cohort of patients with deep supratentorial ICH and concomitant IVH, lower hemorrhage roundness was independently associated with in-hospital mortality. This association remained after adjustment for continuous hematoma volume and the ICH Score. In contrast, hematoma volume showed the strongest association with unfavorable functional outcome at discharge, whereas roundness was not independently associated with functional outcome at discharge. These findings suggest that morphologic irregularity may be more strongly associated with early mortality risk than with short-term functional outcome, which may be influenced by additional factors and includes both severely disabled survivors and deceased patients.

### Morphology beyond volume

Hematoma volume is a well-established predictor of outcome in spontaneous ICH and a core component of the ICH Score [7, 18]. However, volume reflects overall hemorrhage size without describing geometric configuration. Furthermore, the measurement is based on the ABC/2 formula, which may introduce significant bias in the results. In our cohort, roundness showed discrimination comparable to volume in univariable analysis and remained independently associated with mortality in multivariable models.

Previous imaging studies have suggested that hemorrhage shape may reflect underlying bleeding dynamics and mechanical expansion patterns [15]. However, quantitative evaluation of geometric descriptors in spontaneous ICH has been limited. Our findings extend this concept by demonstrating that a reproducible shape metric remains associated with mortality after adjustment for both continuous volume and the ICH Score.

These results indicate that geometric irregularity may capture structural characteristics not reflected by size alone [9]. Reduced sphericity has previously been associated with poorer postoperative recovery in dorsally located spinal meningiomas and with postoperative cranial nerve function and proliferative activity in cranial meningiomas [21, 22]. Moreover, radiomic shape metrics have demonstrated prognostic relevance in subdural hematoma cohorts, including the KEPPRA and PROMISE analyses [5, 6].

### Prognostic information beyond the ICH score

Whether radiomic shape features add information beyond established clinical risk scores is clinically relevant. The ICH Score incorporates hematoma volume in dichotomized form and remains a robust predictor of mortality [17]. Although modeling continuous volume provides a more detailed measure of hemorrhage extent, geometric parameters such as roundness may reflect structural characteristics not fully captured by size alone [15].

The persistence of an association between shape metrics and mortality after adjustment for established predictors suggests partial independence from conventional risk factors [23]. However, the absence of a significant improvement in overall discrimination indicates that radiomic morphology does not replace established clinical scores. Instead, shape-based descriptors may add information beyond established clinical scores. Overall, volume and morphology appear to describe different aspects of hemorrhage geometry [10]. While volume quantifies extent, configuration may relate to spatial expansion patterns and structural complexity.

### Mortality versus functional outcome

Morphologic and volumetric parameters showed different associations with outcome type. Roundness was independently associated with in-hospital mortality but not with unfavorable functional outcome at discharge. This may reflect that functional outcome at discharge is a more heterogeneous endpoint influenced by factors beyond hemorrhage characteristics. Furthermore, combining mRS 5 and 6 may dilute associations more closely related to mortality risk. In an additional analysis using mRS 4–6, neither roundness nor hematoma volume was independently associated with outcome after adjustment for the ICH Score, suggesting that broader functional outcome definitions at discharge may dilute associations more closely related to mortality. In contrast, BGH volume demonstrated strong discrimination for functional outcome. This difference may reflect that early mortality in deep ICH is often related to acute mass effect and rapid neurological decline, whereas functional status at discharge is more closely linked to overall tissue damage and ventricular extension [2, 13, 14, 24]. Accordingly, geometric configuration may be more relevant for early fatal course, while total hemorrhage extent appears more closely related to short-term disability. Given the limited sample size, differences between outcome definitions should be interpreted with caution, as some observed effects may reflect statistical variability rather than true biological differences.

### Clinical and methodological implications

Quantitative shape analysis can be derived from standard non-contrast CT imaging using semi-automated segmentation and predefined geometric descriptors. Restricting the analysis to a limited number of shape features reduces dimensionality and limits the risk of overfitting compared with high-dimensional radiomics approaches. Roundness is directly interpretable and reproducible. In contrast to texture-based features, geometric descriptors are less sensitive to scanner variability and reconstruction parameters, which may support more consistent application across centers [1]. Radiomic shape metrics should therefore be considered as an adjunct to established clinical predictors rather than a replacement.

### Limitations

This study has several limitations. Its retrospective single-center design may limit generalizability, and external validation in independent cohorts is required before clinical translation. Due to the retrospective design, information on withdrawal of care or treatment limitations was not systematically available; therefore, in-hospital mortality could not be differentiated with respect to treatment decisions. Follow-up beyond discharge was not available, and the analysis focused on in-hospital outcomes. The sample size was modest, with 17 mortality events. Although predictor selection was restricted and internal bootstrap validation was performed, some degree of overfitting cannot be excluded. Hematoma segmentation was semi-automated with manual refinement. While conducted according to a standardized protocol, interobserver variability was not formally assessed. The analysis was limited to predefined shape-based features. This approach reduced dimensionality and improved interpretability but did not explore potential contributions from texture- or intensity-based radiomics. Functional outcome was assessed at hospital discharge rather than at 90 days, which is more commonly used in stroke research. Early mRS may not fully reflect longer-term recovery. Finally, the cohort was restricted to deep supratentorial ICH with intraventricular extension, and the findings may not be generalizable to lobar hemorrhages or cases without ventricular involvement.

## Conclusion

In deep supratentorial ICH with IVH, hemorrhage roundness was independently associated with in-hospital mortality after adjustment for hematoma volume and the ICH Score. In contrast, hematoma volume showed a stronger relationship with unfavorable functional outcome. These findings suggest that geometric configuration and volumetric extent reflect different aspects of hemorrhage severity. Shape-based analysis derived from routine CT imaging may add information beyond established predictors. Larger studies with external validation are required to determine the role of geometric descriptors in prognostic modeling of spontaneous ICH.

## Supplementary Information

Below is the link to the electronic supplementary material.

Supplementary file1 (DOCX 380 KB)

## Data Availability

The datasets generated and/or analysed during the current study are not publicly available due to institutional data protection regulations but are available from the corresponding author on reasonable request.
