# Supplementary material for: Radiomic hemorrhage roundness predicts outcome beyond the ICH score in deep intracerebral hemorrhage with IVH
Source: J Neurol. 2026 May 22;273(6):331. doi: 10.1007/s00415-026-13875-1 (PMC13197261; doi:10.1007/s00415-026-13875-1)
Supplement: Supplementary file 1 — Supplementary file1 (DOCX 380 KB) [file 415_2026_13875_MOESM1_ESM.docx]

**Supplementary Table S1.** Baseline characteristics stratified by high versus low BGH roundness (median split). Variables are presented as mean ± standard deviation or number (%), as appropriate.

| Variable | High roundness (n = 25) | Low roundness (n = 25) | p-value |
| --- | --- | --- | --- |
| BGH roundness | 0.78 ± 0.05 | 0.66 ± 0.06 | <0.001 |
| Age (years) | 70.8 ± 13.4 | 69.2 ± 13.0 | 0.678 |
| Sex, male (%) | 14 (56%) | 15 (60%) | 1.000 |
| Admission GCS | 8.0 ± 4.8 | 6.3 ± 3.8 | 0.157 |
| ICH Score | 2.7 ± 1.1 | 2.9 ± 0.8 | 0.387 |
| BGH volume (cm³) | 23.8 ± 20.1 | 31.1 ± 37.5 | 0.399 |
| IVH volume (cm³) | 17.0 ± 22.7 | 19.9 ± 24.2 | 0.655 |


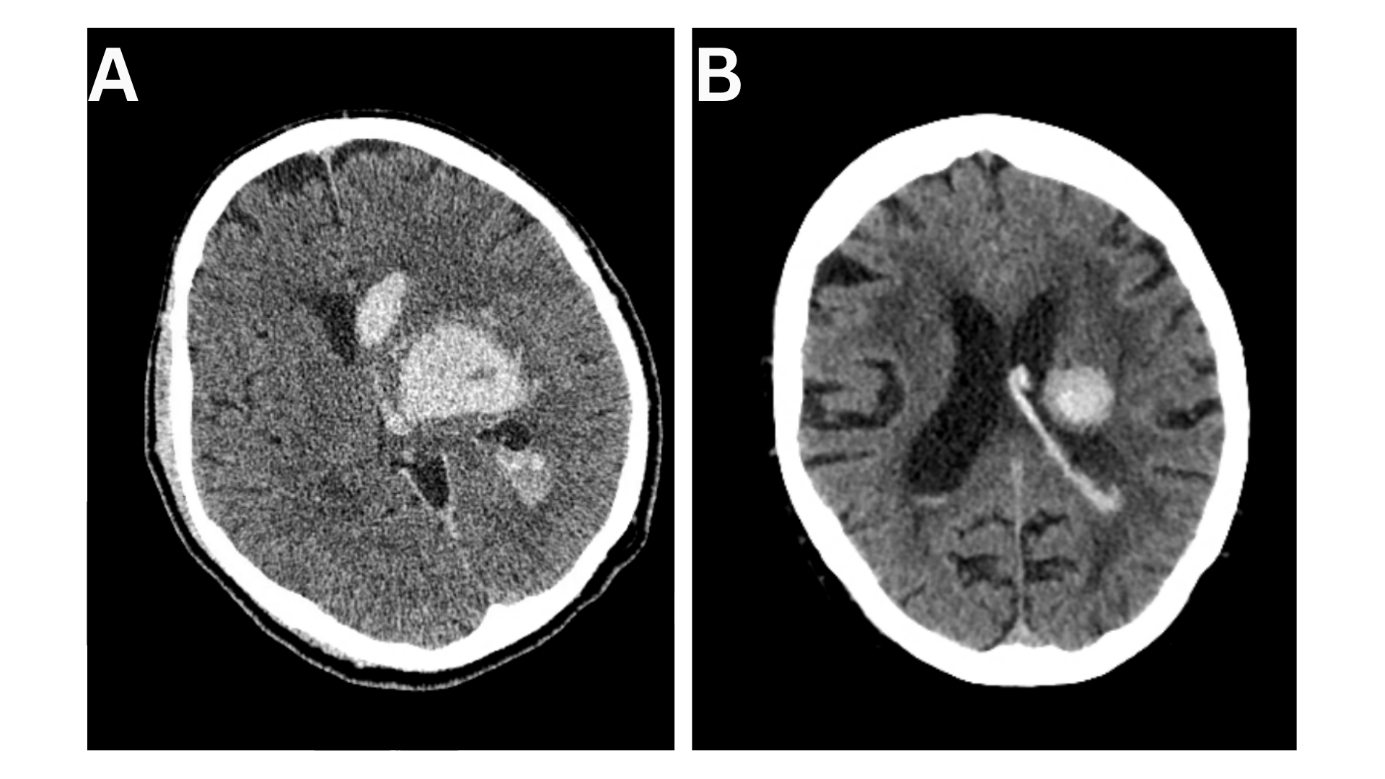


**Supplementary Figure S1.** Representative examples of basal ganglia hemorrhages on axial non-contrast CT illustrating differences in geometric configuration. (A) Hematoma with low roundness (0.64), demonstrating an irregular morphology. (B) Hematoma with high roundness (0.85), demonstrating a more spherical configuration. Roundness reflects the similarity of the segmented hemorrhage to a sphere, with values closer to 1 indicating a more regular shape.
